# Decoding time from space: A review of the complication clock and its representation of temporal experience

**DOI:** 10.3758/s13423-026-02951-2

**Published:** 2026-07-07

**Authors:** Nathan Thomas Han, Michael B. Steinborn, Liyu Cao

**Affiliations:** 1 Department of Psychology and Behavioural Sciences, Zhejiang University, Hangzhou, China; 2 The State Key Lab of Brain-Machine Intelligence, Zhejiang University, Hangzhou, China; 3 School of Psychological and Cognitive Sciences, Peking University, Beijing, China; 4 Department of Psychology (III), Julius-Maximilians-Universität Würzburg, Würzburg, Germany; 5 Zhejiang Key Laboratory of Neurocognitive Development and Mental Health, Zhejiang University, Hangzhou, China

**Keywords:** Time perception, Sense of agency, Temporal binding, Libet clock, Temporal experience

## Abstract

The complication clock, originally introduced by Wilhelm Wundt, remains a pivotal method in experimental psychology for probing the subjective timing of events. By localising the position of a moving pointer, one can objectively measure when someone perceives an event to have occurred. The method therefore maps temporal judgments via a spatial representation of time on the basis of a moving pointer. Although it provides a unique tool for capturing otherwise unobservable phenomena, the method also raises critical conceptual and methodological challenges. In this article, we provide a historical account of the research, from early complication experiments on sensory processing through Libet’s (Libet et al., 1982) repurposing for volition research to present-day investigations of sense of agency, affect, and perceptual awareness. We then discuss the measurement structure of the clock, illustrating how the spatial nature of the clock introduces systematic distortions to time reports and examining conditions under which these distortions can be identified and controlled. We further show that the method carries an implicit commitment to serial-discrete temporal order, constraining the range of cognitive phenomena that the clock is able to investigate. These analyses can help to inspire greater methodological and conceptual sensitivity for future investigations into our subjective timing of events.

## Introduction

The complication clock, commonly referred to as the Libet clock (Libet et al., 1982), consists of a clock face and a pointer and allows for the measurement of *when* a participant perceives an event to occur. Wilhelm Wundt originally introduced the term “complication” to capture the interaction between sensory stimuli and cognitive processes such as attention, resulting in subjective time reports that often differed from objective measurements (Wundt, 1873).

Early complication clock experiments primarily focused on how factors like attention, the nature of stimuli, and individual variability influenced sensory processing. Over time, the applications of the complication clock extended beyond sensory processing. From the 1980 s onwards, Benjamin Libet revolutionised its use to explore the temporal dynamics of volition (Libet et al., 1982). Libet’s experiments used the clock to synchronize three key elements: unconscious brain activity (measured by the readiness potential in EEG recordings), participants’ subjective awareness of their intentions, and their observable physical actions. He demonstrated that a neural signal for action preceded conscious awareness of deciding to act, sparking significant debates about free will and volitional control.

This evolution of the complication clock marked a transition from studying reactions to external stimuli towards probing the internal mechanisms of cognition. The complication clock has now been widely adopted across disciplines, expanding its applications to encompass cognitive processes (Hogendoorn et al., 2007; Kang et al., 2017), emotion (Franikowski et al., 2021), and even psychopathologies (Doñamayor et al., 2018; Yabe et al., 2019). However, despite its versatility, there continues to be debate over the precision and reliability of its measurements.

The aim of the present review is to critically examine the complication clock, which is fundamentally a tool used to extract the subjective timing of events using a spatial measure. Although this presents a convenient way of measuring phenomena that cannot be directly observed, its spatial nature leads to problems that span across all studies using the clock. We first introduce the complication clock, followed by a history of the studies using the clock. One of the things we wish to emphasise is the shift from primarily studying the perception of external stimuli to studying the timing of cognitive processes. We then detail the mechanisms involved and highlight potential practical and theoretical issues with the complication clock. In fact, since the introduction of the Libet studies (Libet et al., 1982), there have always existed debates regarding the reliability and validity of the complication clock, though they tend to be centred within the specific research program using the clock. We argue that the complication clock is a spatial representation of our psychological experience of time and therefore assumes an isomorphism between space and our psychological experience of time. However, by modelling psychological time on one-dimensional perceived space, the complication clock fundamentally relies on a conception of time perception that has recently come under criticism (Guo et al., 2025; Robbe, 2023; Salet et al., 2022). Finally, we end by discussing how the practical and theoretical issues could be addressed in future use of the complication clock. In particular, we focus on how modern theoretical questions that are inherently more dynamic could also be addressed by the complication clock.

### The complication clock

**Fig. 1:**
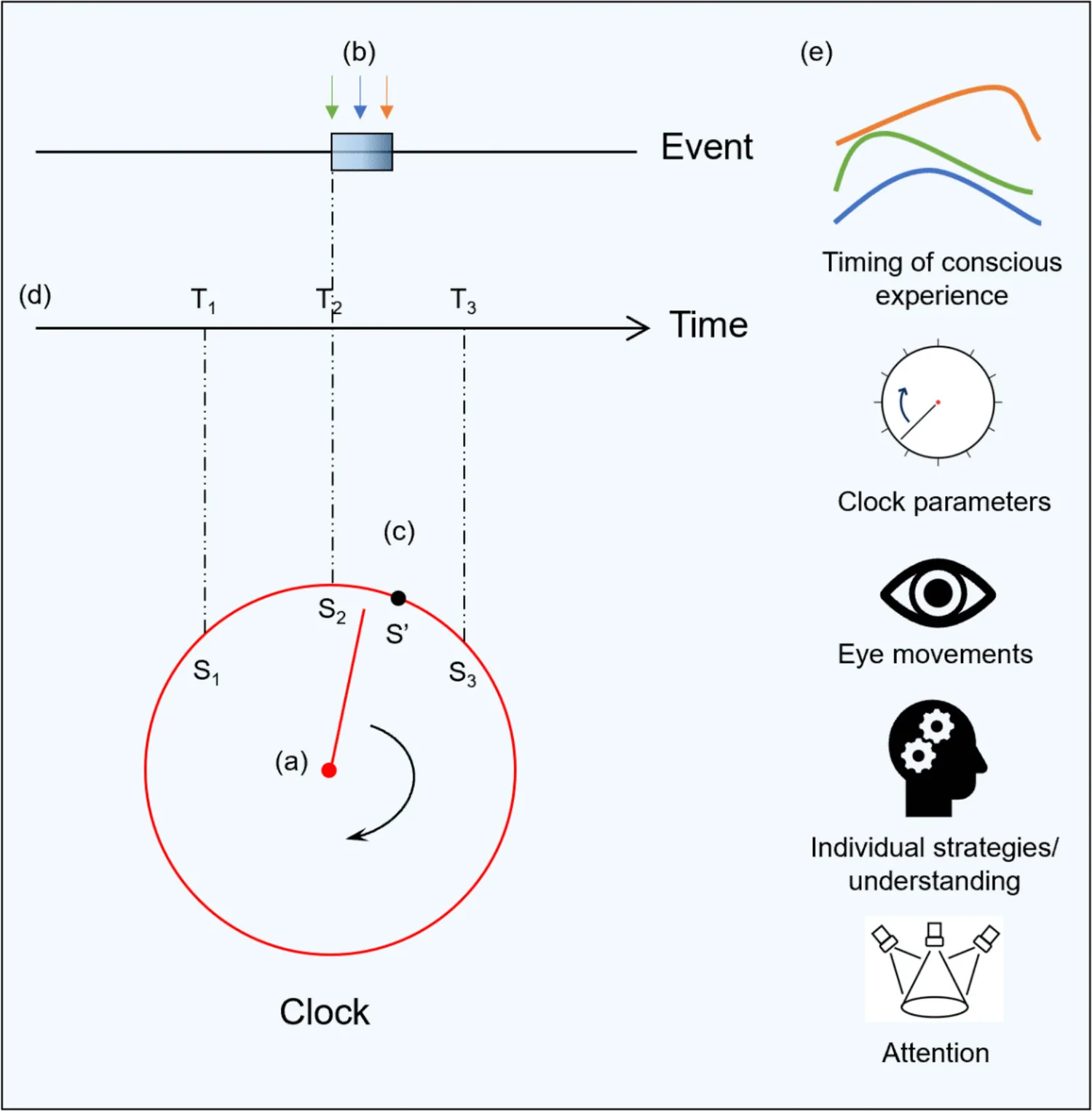
Anatomy of a time report obtained with the complication clock. Participants observe a clock face with a rapidly spinning pointer (**a**) and an event occurs (**b**), which can be internal such as motor intention or external such as a keypress. Highlighted in red are the elements that participants actually see during the testing (the clock face and pointer). The event onset is at time T2. They then give a time report corresponding to where the pointer was when the event occurred, here marked by S’ (**c**). In this case, the reported S’ is later than the actual event onset. The complication clock method maps time to space (**d**), as subjective timing is derived from spatial locations. For example, locations S1, S2, and S3 on the clock correspond to three distinct, objective time points T1, T2, and T3, respectively. The time report is influenced by many factors. For example, an event occupies a time period rather than a time point (e.g., a keypress usually lasts more than 200 ms). Individual differences in the perceptual centre of the event (denoted by the different coloured arrows on (**b**) can lead to a difference in the time report of the event. Numerous factors can affect participant time reports such as the clock parameters (pointer speed, etc.), eye movement behaviour, attention, individual differences in task strategies/understanding, and latencies involved with the conscious experience of separate stimuli (**e**)

The complication clock is used to measure the subjective timing of an event. Despite the many research questions featuring the clock, the basic elements of the task remain identical. Participants in the complication clock task observe a clock face with a pointer. The pointer rapidly spins around the clock face. The to-be-reported event (e.g., a sound or a keypress) occurs while the pointer is spinning. Participants then report the time of when the event occurred by reporting the pointer position at the event occurrence. As an example of a typical trial (Fig. 1), a participant is asked to report the keypress time. While the pointer is spinning, the participant decides when to perform a keypress. After the keypress, the participant then verbally reports 10 as the keypress time (or moving the pointer to the 10-min position; the clock face is divided into 60-min marks as in reality). Nearly all studies using the complication clock follow this basic structure, with variations mainly in the clock parameters (pointer speed, clock size etc.) and the event-of-interest being reported.

The complication clock is essentially a method that maps time to perceived space with the rotating pointer as a medium (Fig. 1). With the unfolding of time, the pointer moves through the clock face. Therefore, different spatial locations on the clock face correspond to different time points. The timing of an event is reported as a spatial location on the clock face. The time report (i.e., the reported pointer position at the time of event onset) is always referenced for data analysis, either to the actual timing of the event itself or to another event (e.g., brain activity; Libet et al., 1982, Libet, Gleason et al., 1983). In the former case, a report error is calculated as the difference between the reported pointer position and the actual pointer position at the event occurrence. The report error is then converted to time as the rotation speed of the pointer is known. Therefore, the report error is a signed value, with positive values indicating that the reported time is in the future of the actual time (positive errors) and negative values indicating the opposite (negative errors). An implicit assumption is that the time report is more or less accurate. That is, the absolute value of the report error cannot exceed half of the period of the pointer rotation. For example, if a keypress is made when the pointer is at the 30-min position and the participant reports the 40-min position, it is assumed that report error is 1/6 (not −5/6, 7/6 or any other theoretically possible values) of the period.

The complication clock rests on two presumptions. The first concerns the measurement procedure: the pointer and its uniform motion serve as a spatial proxy for the ongoing flow of time, and participants localise the pointer to indicate when they perceive an event to have occurred. Because localisation of a moving object is subject to perceptual biases (e.g., representational momentum, attentional distribution, oculomotor behaviour), the time report is a combination of the event, the localisation, and the biases operating between the two (Cao, 2024). Here, we provide an example illustrating the different biases that can be involved in the construction of a time report.

For example, an event-onset time is at T_2_, which corresponds to spatial location S_2_ (Fig. 1). Spatial biases can affect time reports. The reported event onset (S’, Fig. 1c) is in the future of the actual event onset (S_2_) due to a phenomenon known as representational momentum, which involves the memory of the location of an object being mistakenly displaced forward in the direction of the object motion (Joordens et al., 2002). The operation of representational momentum has sometimes been attributed to prediction of the moving object, which is here facilitated by the uniform motion of the pointer. Although event onset ostensibly occurs at a discrete point in time, the event may in fact occupy a time period (e.g., a keypress usually lasts for more than 200 ms; Fig. 1b). Different participants may therefore psychologically register the occurrence of the event at different moments (denoted by the different coloured arrows *green, blue, orange*), corresponding to the perceptual centre, or p-centre, of the event (Morton et al., 1976; Scott, 1998), thus leading to interindividual differences in time reports. This example only mentions a handful of mechanisms that go towards the construction of a time report, though there are numerous more (Fig. 1e). We detail these mechanisms in the later section “The mechanisms involved in the complication clock method”.

Secondly, the complication clock assumes an isomorphism between one-dimensional perceived space and our psychological experience of time. For example, a participant might give time reports for two separate events in two different conditions, events *X* and *Y*, at spatial positions on the clock *t* and *t + 1* (with each unit representing a tick mark on the clock face). Given that the rotation speed of the pointer is known in advance, the pointer position can thus be converted to time. After the conversion of the spatial position to time, one might say “participants perceived event *X* to occur 20 ms before event *Y*”. Importantly, it can be said that the complication clock directly equates our time perception of events with space. This presumption is problematic because it facilitates and biases a seriality to the way we conceive of cognitive processes. The linearity of the pointer motion and conception of time through the spatial representation means that at face value, the complication clock is more comfortable accommodating theories of cognition and behaviour that operate serially as opposed to in parallel. This can present an issue as theories of cognition and behaviour are increasingly shifting away from simple laboratory settings where phenomena occur linearly within a simple stimulus-response chain and towards more dynamic conceptions where cognitive and behavioural processes may occur in parallel (Cisek & Green, 2024; Gomez-Marin & Ghazanfar, 2019).

### An overview of the research questions addressed by the complication clock

**Fig. 2:**
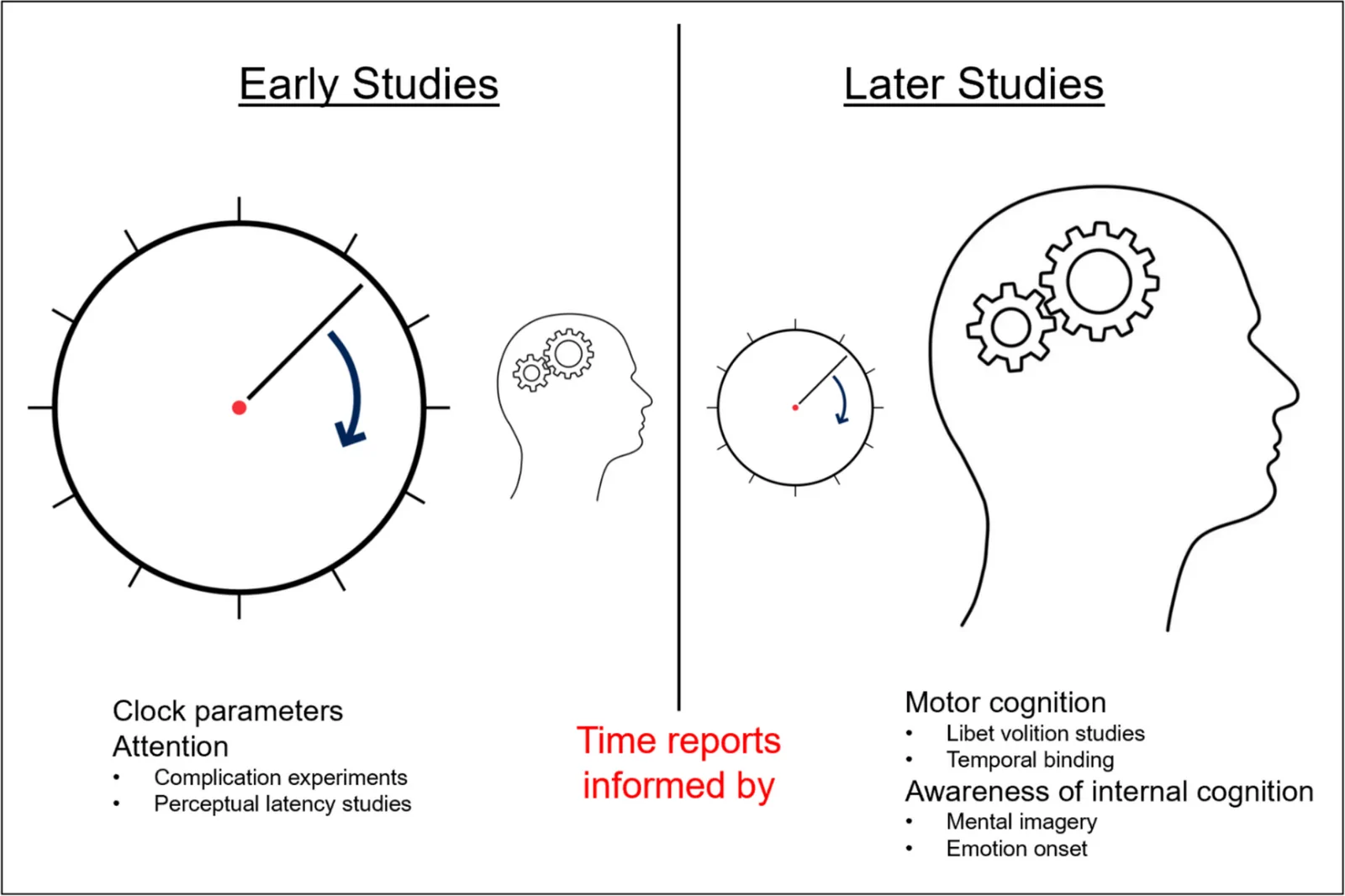
A shift in focus from early versus late studies using the complication clock. With Wundt and his contemporaries’ early complication experiments until the 1980 s, studies using the complication clock tended to focus on the nature of sensory processing of external stimuli. Furthermore, much of the focus was on how clock parameters, such as pointer speed, and attention affected time reports. From the 1980 s onwards, Libet repurposed the complication clock to investigate volition. Since then, time reports have been used to represent more complex internal phenomena such as the awareness of affect onset or motor cognition. Time reports are the common observable across both cases. What differs is the epistemic object that the report is taken to indicate

Originally developed by Wundt, the complication clock was instrumental in studying elementary sensory processing and the integration of sensory inputs with attention. Over time, its usage expanded, with a focus shifted towards internal processes such as volition and conscious intention (Fig. 2). While its applications have now extended to studying volition, emotion, attention, and somatosensory processing, its core principle remains: measuring subjective time perception in relation to objective events. Here, we detail the different lines of research that have used the complication clock as well as how the results attained by the clock use fit in with the rest of the literature.

### Sensory processing

#### The complication experiment

Wilhelm Wundt introduced the complication experiment to investigate how sensory inputs are processed and transformed into subjective temporal judgements. According to Wundt, the time report in this method was not a faithful representation of the pointer position, but something that emerges from the fusion of the sensory input and attentional state of the participant. The term “*Komplikation*”, which Wundt derived from the philosopher Johann Friedrich Herbart, was meant to capture the idea that sensory inputs are “complicated” alongside cognitive processes to form higher-level percepts. An early notable study is that of von Tchisch (1885), who used the complication experiment to investigate how different event modalities and the number of stimuli influenced the time reports. While the stimulus modality had no significant effect, an increase in the number of stimuli led to a clear rise in positive errors. Another study by Pflaum (1900) examined how pointer speed affected time reports. He found that faster pointer speeds led to greater positive errors. Building on this, Geiger (1903) showed that both pointer speed and practice influenced time reports, with speed having a stronger effect. Slower speeds produced negative errors, while faster speeds led to positive errors. Notably, extended practice increased positive errors rather than improved precision.

#### Perceptual latency

One of the key topics within sensory processing is perceptual latency, the time between stimulus onset and perception. For example, Sanford (1971, 1974) explored the relationship between stimulus intensity and both simple reaction time and time reports. It was known that louder sounds led to faster responses (Chocholle, 1940). Sanford sought to investigate whether the stimulus intensity-reaction time relationship could simply be explained by the time it took to detect the stimulus (that is, perceptual lag) or by the time it took to initiate a response. Participants heard background white noise of 60 dB and observed a rotating pointer on a clock face while also keeping their finger pressed on a switch key (Sanford, 1971). After a period of at least one pointer rotation, participants heard an auditory stimulus with intensities of 62, 63, 67, or 78 dBs, separated amongst separate blocks. Participants performed two tests in separate blocks (experiment 1) or in the same block (experiment 2). In the first test, participants released the key as soon as they detected the auditory stimulus (*reaction time test*). In the second test, participants were told to judge when the stimulus appeared by moving the pointer to the stimulus onset position (*time report test*). Therefore, the reaction time test assessed simple reaction time while the pointer test ostensibly assessed the perceptual lag of the stimulus registration. The results from both experiments were conceptually identical: both reaction time and time reports decreased as stimulus intensity increased. However, the slope of reaction time decrease was steeper than the slope of time report decrease. Based on these results, Sanford concluded that there is an intensity-dependent lag between detecting a stimulus and initiating a response to it, indicating that much of the effect of auditory intensity on reaction time can be explained in terms of response factors rather than perceptual latency. The results were replicated in Sanford (1974). A later study using the complication clock by Wolpe et al. (2013) also found time reports for loud sounds were earlier compared to soft sounds. Therefore, consistent behavioural results were obtained across studies indicating that sounds with increased intensity indeed shortened auditory perceptual latency. Several studies using EEG have also reported that different stimulus intensities can change the latencies associated with different components of the event-related potential (ERP) (Brisson et al., 2007; Connolly & Gruzelier, 1982). Whether this translates to changes in the conscious experience of stimuli that timing reports are based on, however, still needs further research.

Seifried et al. (2010) extended the results of the previous studies by investigating if temporal preparation could shorten perceptual latency. In their study, participants gave time reports to target tones that were preceded by a warning tone, with the time between the target tone and warning tone varying between blocks to allow participants to build up an expectation and prepare for the target tone (i.e., the constant foreperiod paradigm (Näätänen et al., 1974; Niemi & Näätänen, 1981)). The authors demonstrated shorter reaction times to the target tone when the delay between the tone and the preceding warning tone was short, in comparison to a long delay. At the same time, the time report of the target tone was also earlier for short delays compared to long delays. It was therefore concluded that increased temporal preparation shortens the perceptual latency of a tone. Several follow-up studies by the same research group have also demonstrated that temporal preparation can decrease perceptual latency using different experimental methods such as EEG (Seibold et al., 2011; Seibold & Rolke, 2014). For example, Seibold et al. (2011) used the N2 component, an index of perceptual processing, and investigated whether its latency was shortened by increased temporal preparation. Similar to Seifried et al. (2010), they found that increasing temporal preparation reduced the latency of perceptual processing via a decreased N2 component latency.

Finally, the complication clock has also been used to investigate sensory processing in patients with Huntington’s disease (Mirallave et al., 2017). Huntington’s disease is a neurodegenerative disorder characterised by involuntary movements such as chorea (involuntary and unpredictable movements), slowing of movements, and progressive cognitive decline (Anderson & Marder, 2001; Roos, 2010). EEG studies of patients and asymptomatic gene carriers have shown delayed latencies and decreased amplitudes in certain components of ERPs (Abbruzzese & Berardelli, 2003). The delayed latency and decreased amplitude of the ERP components have often been attributed to either cognitive impairment (Legrain et al., 2011) but may also be due to delayed sensory processing. Mirallave et al. (2017) used the complication clock to investigate whether patients’ dysfunction of ERP components was associated with either cognitive impairment or delayed sensory processing. On *Rest* trials, patients and healthy controls gave time reports for the onset of electrical and thermal stimuli. On *React* trials, in addition to the time report, participants were required to perform a reaction time test in which they made a fist with their right hand as quickly as possible when they perceived the stimulus. Compared to healthy controls, patients showed a delayed N2 component (an ERP component shown to be related to several processes including cognitive control, attention, and novelty (Folstein & Van Petten, 2008)) as well as reaction times. Furthermore, both groups showed later time reports for the *React* trials compared to *Rest* trials (see also Yabe & Goodale, 2015). However, patients also showed later time reports compared to healthy controls in the *React* trials when using thermal stimuli. Based on these results, Mirallave et al. concluded that patients showed deficits in detecting stimulus salience rather than cognitive decline.

### Volition and sense of agency

Wundt and his contemporaries had already taken into account the fact that time reports were inaccurate. The reason is that the objective occurrence of an event must pass through the complication of the perceptual system (Wundt, 1873). Most modern studies that use the complication clock implicitly assume time reports can more or less accurately capture subjective timing of the event-in-question and rarely cite the early complication studies.^1^ This is likely because report errors can be easily verified with external events, whereas this is not the case for internal phenomena. In the 1980 s, the complication clock assumed a new role when Benjamin Libet adapted it to explore volition and conscious intention. Although originally named the complication clock, the clock stimulus is perhaps now better known as the Libet clock. Apart from the Libet volition studies (Libet et al., 1982, Libet, Gleason, et al., 1983), the clock has been most used in studies of temporal binding, a phenomenon which has been argued to be an implicit measure of sense of agency (Beck et al., 2017; Moore & Obhi, 2012; Wohlschläger et al., 2003).

#### The Libet volition experiment

In the free-will and volition experiments most associated with Libet, participants observed a rotating pointer and spontaneously performed a finger/wrist movement. They then reported the precise moment when they became aware of their intention to move. It was found that onset of slow-forming brain activity called the ‘readiness potential’ (Kornhuber & Deecke, 1965) tended to precede the reported time of intention by about 300 ms or more. The results were interpreted as supporting the hypothesis that the brain initiates actions before we become consciously aware of our intention to act, therefore questioning the intuitive belief that conscious mental states cause behaviour. This interpretation by Libet was based on his notion of *backwards referral* (Libet et al., 1979)*,* in which he argued that the subjective experience of sensory events lagged about 500 ms behind stimulus detection, but is subjectively referred back in time to the moment of physical occurrence.

Though Libet is primarily known for his famous “free will” experiments in the 1980 s, the studies were also a continuation of his earlier work on how conscious experience was produced by brain activity. For example, Libet et al. (1964) showed that a long stimulus train stimulating the somatosensory cortex needed to last at least 500 ms to produce a conscious sensation. However, the authors also found that short stimulation to the skin (as short as 50–100 ms) was enough to produce a conscious sensation. To address the possibility that the first result was due to artificial cortical stimulation, Libet et al. (1979) conducted further experiments showing a similar result when a long stimulus train was applied to the medial lemniscus, a group of nerves that leads signals from the periphery (such as with the second result of the skin in Libet et al. (1964)) to the somatosensory cortex. This result showed that even when the elicited signal reached the somatosensory cortex indirectly, one still needed a long-lasting stimulus train to produce a conscious sensation. Finally, Libet et al. (1979) also investigated the temporal relation between a continuous stimulation to the somatosensory cortex/medial lemniscus and a peripheral stimulus that was delivered simultaneously with the start of the continuous stimulation. The results showed that cortical stimulation was experienced as delayed compared to the peripheral stimulus. However, peripheral stimulation and the continuous stimulation delivered to the medial lemniscus was experienced as simultaneous. This led Libet and colleagues to put forward the idea of *backwards referral*, wherein the timing of a conscious sensation is back-dated to the time of the onset of the stimulus (Libet et al., 1979; Pockett, 2002), and became the basis of his famous studies on free will using the complication clock. However, this notion of backwards referral was highly controversial^2^ (Churchland, 1981; Gomes, 1998, 2002a; Pockett, 2002; van de Grind, 2002).

For example, Pockett (2002) argued that the direct cortical stimulation was a highly abnormal method of producing a conscious sensation. Normal stimulation, such as the peripheral stimulation of the skin, would arrive at the somatosensory cortex in a spatially organised pattern of neural activity first originating from stimulation of peripheral receptors. However, direct cortical stimulation could not reproduce such a spatial pattern of neural activity. Therefore, no concrete conclusions could be made about how long it took for “normal” stimuli to produce a conscious sensation.

Another relevant point, which was made by Gomes (2002b), is what he called the “irrevocable decision to act now” and is related to Searle’s (1983) notion of intention-in-action. The idea is simply that we often perform voluntary actions without any preceding conscious intention of doing so. An example used by Gomes (2002b) and taken from Searle (1983) is of someone who is thinking about a difficult philosophical problem and suddenly decides to walk across the room. In such an example, the walking is clearly conscious and voluntary, but there is no conscious intention preceding the action.

The results of the Libet experiments also led to widespread debate in the neuroscience and philosophy communities about the nature of free will (Dennett & Kinsbourne, 1992; Dominik et al., 2024). Studies have also used the Libet volition paradigm to investigate deficits of volition in various disorders such as schizophrenia (Pirio Richardson et al., 2020) and Parkinson’s disease (Giovannelli et al., 2022; Tabu et al., 2015). More details regarding the Libet studies and the debates surrounding his results can be found in the excellent review by Dominik et al. (2024).

#### Temporal binding

Temporal binding is the phenomenon that an action and its sensory outcome are perceptually attracted towards one another in time relative to when either event occurs in isolation (Cao et al., 2020; Haggard et al., 2002; Klaffehn et al., 2021; Ruess et al., 2017; Wolpe et al., 2013). In the temporal binding experiment, participants view a rotating pointer and make time reports about the keypress or sound onset. In *baseline* conditions, the keypress or sound appears in isolation and time reports are made about the individual event. In *instrumental* conditions, the keypress is followed by a sound after a delay of ~250 ms, with time reports made for either one of the two events. Temporal binding experiments usually demonstrate later reported times for keypresses in the *instrumental* condition compared to the *baseline* condition and earlier reported time for sounds in the *instrumental* condition compared to the *baseline* condition. These results ostensibly demonstrate that the perception of action-outcomes is temporally pulled towards one another. Accordingly, temporal binding can be separated into action binding (the temporal shifting of action) and outcome binding (the temporal shifting of the resulting outcome). The temporal binding effect has been studied under many different contexts. Examples include how agency is affected by different forms of mental illness as well as other novel contexts such as applied human-computer interface research (Bergström et al., 2022; McEneaney, 2009) including virtual reality (Cornelio et al., 2020) and mid-air interfaces (Cornelio Martinez et al., 2017).

The temporal binding effect has also been studied with another popular method called the interval estimation method (Engbert et al., 2008; Humphreys & Buehner, 2010; Kong et al., 2024). In experiments of interval estimation, participants explicitly report the elapsed time between a voluntary action and an effect. This is compared with another condition wherein a cue or an involuntary action replaces the action. Studies using the interval estimation method have claimed that it allows for the study of temporal binding without some of the confounds that come with using the complication clock task (Siebertz & Jansen, 2022). For example, a study by Siebertz and Jansen (2022) directly compared the use of the complication clock and interval estimation method in measuring temporal binding. As both studies ostensibly measured temporal binding, they hypothesised that there would be a positive correlation between the two measures. A Bayes factor analysis showed moderate evidence for the binding measure of the two experiments to be uncorrelated. They found the reliability of the binding measure for the complication clock paradigm to be higher, suggesting that interval estimation introduces substantial measurement error. Furthermore, it can be argued that the complication clock task and the interval estimation task measure different aspects of time perception, with the first task measuring *when* different events occur – with perceived duration inferred – while the other involves an estimation of duration. The difference between the two types of time perception is not trivial, with evidence showing distinct neural correlates between the two (Coull & Giersch, 2022).

In addition, temporal binding has been investigated using an auditory timer method (Muth et al., 2020). To address the spatial confound of the complication clock, Muth et al. (2020) had participants listen to a sequence of letters which was used to anchor their temporal judgements regarding the action and its subsequent visual effect. Across four experiments, Muth et al. found temporal binding effects for most of their experiments. Finally, another method is the event-anticipation method (Buehner, 2012; Roth et al., 2023). In this method, a time interval (e.g., 500 ms) is initiated with a voluntary keypress (*self-cause*), a lever press initiated by a detached machine (*other-cause*), or with a visual cue (*passive*). At the termination of the time interval, a visual stimulus may or may not appear. Participants are required to perform a keypress in anticipation of the visual stimulus onset. Anticipation keypress times were shorter in the self-cause and other-cause conditions than in the passive condition, indicating that temporal binding in this paradigm depends mainly on recognising causation.

The studies mentioned above show that the temporal binding effect is fairly robust, even across different methodologies. However, although temporal binding has traditionally been argued to be an implicit measure of sense of agency, this interpretation has been challenged in recent years*.* A growing body of research has proposed alternative accounts for temporal binding using both the complication clock (Cao, 2024; Schwarz & Weller, 2023) and interval estimation methods (Kong et al., 2024). For example, several studies have shown that the temporal binding effect can be found without participant sense of agency or intentionality (Gutzeit et al., 2023; Kirsch et al., 2019; Schwarz et al., 2019). Recently, several studies have also argued that the temporal binding effect can be explained (at least partially) with factors such as attention (Cao, 2024; Chen et al., 2025; Schwarz & Weller, 2023; Wang et al., 2025) and temporal prediction (Kong et al., 2024).

### Other internal processes using the complication clock

Beyond the investigation of when intentions enter awareness and how actions and their effects are linked in time, the complication clock has also been applied to other internal processes. Here, the target of timing is a moment within ongoing experience itself. The method is therefore used in cases where the relevant event cannot simply be identified with stimulus onset or an overt response, but must be located via the time report. This includes studies on affective experience, decision awareness, voluntary processes in relation to respiration, and attentional shifts. The common point is the temporal localisation of an internally accessible event.

#### Emotion

The complication clock has been used to investigate emotion (Franikowski et al., 2021) and the effect of emotional states on volition and sense of agency (Rigoni et al., 2015; Yoshie & Haggard, 2013). Franikowski et al. (2021) used the complication clock to investigate the nature of affective experience. For example, people seem to be reflexively disgusted at the sight of rotten food (Junge & Reisenzein, 2013). According to some emotion theorists, having an affective reaction to an object requires a substantial amount of information processing to first recognise the object then to evaluate it as either positive or negative (Storbeck et al., 2006). This hypothesis is known as the *semantic primacy hypothesis* of emotion (Storbeck et al., 2006). An alternative hypothesis of emotion generation is known as the *affective primacy hypothesis* (Öhman & Mineka, 2001). According to this view, some emotions can be triggered by stimuli via a “noncognitive” pathway that avoids the need for higher-level cognitive processing. Franikowski et al. (2021) sought to use the complication clock (a rectangular design in their study) to test which hypothesis was more plausible. In two experiments, participants were presented with pleasant or unpleasant pictures while they observed a moving bar. In separate blocks of trials, participants either reported when they experienced the pleasant/unpleasant feelings evoked by the pictures or when they recognised the object depicted in the picture by giving a time report of when the mental events occurred. In both experiments, participants reported earlier times for object recognition compared to the feeling of affect. Furthermore, the two latencies were positively correlated across participants. Franikowski et al. therefore concluded that the findings supported the *semantic primacy hypothesis*.

These findings would also seem to replicate studies that have investigated the same question using temporal order judgements and simultaneity judgements (Reisenzein & Franikowski, 2022). A reaction time task, in which participants performed saccades to either pleasant/unpleasant images or images that contained an animal (thus requiring object recognition), also supported the semantic primacy hypothesis (Nummenmaa et al., 2010). Lastly, studies that have used subliminal perception (Lähteenmäki et al., 2015) and the continuous flash suppression paradigm (Hedger et al., 2015) also show that object recognition is necessary before affect onset. Thus, the results found in Franikowski et al. (2021) would seem to support numerous converging studies.

#### Perceptual decision making

Kang et al. (2017) used the complication clock to investigate perceptual decision making. It has been argued that perceptual decision making can be represented by a stochastic process of evidence accumulation until a threshold is reached, i.e., the drift diffusion model (Gold & Shadlen, 2007; Ratcliff & McKoon, 2008). For example, when asked to decide the net direction of motion for a group of moving dots (e.g., moving either left or right), participant choices and reaction times can be explained by a model in which evidence is accumulated until it reaches one of two thresholds indicating the direction in which the dots are moving (Mulder et al., 2012). Kang et al. (2017) conducted two experiments to investigate the question of when this unconscious process of evidence accumulation reached conscious awareness. Participants observed a random dot motion stimulus while simultaneously observing a rotating pointer (both stimuli were located within the same space centrally at the screen). After a random duration, the random dot motion stimulus disappeared while the central clock remained, and after a delay, a tone appeared signalling participants to indicate the perceived motion direction. Participants then gave a time report regarding the time of their being aware of their decision. Their second experiment was largely similar to their first experiment, although instead of waiting for the dots to disappear, participants could report their decision as soon as they were ready to do so. The results showed that participants gave earlier time reports of their decision time when the direction of the dots was easier to perceive. The authors thus concluded that conscious awareness of a decision arises when the evidence accumulation process reaches a threshold, with easier decisions crossing the threshold more quickly.

Generally, there is large body of research supporting the evidence accumulation or “sequential sampling” model of perceptual decision making (Cisek et al., 2009; Grossberg & Pilly, 2008; Smith & Ratcliff, 2004). There also exist several neural studies based on the same evidence accumulation framework that have complemented the findings of Kang et al. (Pereira et al., 2021; Tagliabue et al., 2019). For example, using a tactile stimulus at around near-threshold level for detection, Pereira et al. (2021) found that detection of the tactile stimulus elicited neuronal responses at the posterior parietal cortex resembling an evidence accumulation process. In another study, Tagliabue et al. (2019) used the centro-parietal positivity, an ERP component argued to be a neural correlate of sensory evidence accumulation (Kelly & O’Connell, 2013; Loughnane et al., 2016), and showed that its magnitude corresponded to the strength of participant percept when they had made their decision. The findings of these studies suggest that the results of Kang et al. (2017) converge with the rest of the research area.

#### Respiratory influences on voluntary action

Recent research on brain-body interactions has seen an interest in the role of interoceptive signals such as respiratory or cardiac signals on brain processing (Ito et al., 2014; Kluger & Gross, 2021). Park et al. (2020) used the complication clock to investigate the role of respiratory influences on voluntary action. In their experiment, participants performed a keypress then reported via the pointer when they felt the urge (or intention) to do the keypress. During the experiment, a continuous respiratory signal was collected as well as an EEG to look at readiness potentials. The authors found that participants were more likely to press the key during the expiration phase of the respiratory cycle, demonstrating that voluntary action was coupled with the respiratory cycle. Furthermore, they found that the amplitude of the readiness potentials was more positive during the inspiration phase of the respiratory cycle.

Park et al. (2022) extended their previous results by also examining the role of respiratory influences on mental imagery. In addition to the keypress, participants also engaged in mental imagery wherein (a) they imagined themselves pressing a key to stop the pointer (motor imagery), and (b) they imagined the pointer stopping by itself, without any movement (visual imagery). Participants then gave a time report regarding the time they engaged in the mental imagery. Similar to the previous paper, Park et al. (2022) found that participants tended to engage in both motor and visual imagery during the expiration phase of the respiratory cycle. They also found readiness potentials leading up to the point in which participants reported they had engaged in the mental imagery.

In both studies, Park and colleagues found that participants tended to engage in these actions during the expiration phase of the respiratory cycle. These studies are part of a growing body of research investigating the interaction between interoceptive signals (e.g., respiratory and cardiac signals) and brain/psychological phenomena (Ito et al., 2014; Kluger & Gross, 2021). Numerous studies have also investigated the role of respiration and cardiac phase on motor activity (Al et al., 2023; Johannknecht & Kayser, 2022; Mussini et al., 2024). For example, across a series of six tasks testing sensory detection, discrimination, and short-term memory, Johannknecht and Kayser (2022) demonstrated that participants tended to give responses during the expiration phase of the respiratory cycle. Overall, the results found in Park et al. (2020, 2022) would seem to corroborate those found across the body of research investigating body-brain interaction. However, a recent review by Caparco et al. (2025) claimed that the classification of the cardiac phase was inconsistent across studies and warned that such an issue may also occur in studies investigating the respiratory phase. The study therefore suggested that there was a need for standardisation of the process for classifying for both respiratory and cardiac signals.

One potential issue, similar to the issue of timing other internal phenomena such as intention, is that mental imagery (e.g., in Park et al., 2022) may not actually occupy a single point in time but instead may span across time. This can lead to systematic biases in pointer localisation depending on how participants make their timing reports regarding their mental imagery. However, this issue is not intractable in the context of locating the imagery within the respiratory cycle as the respiratory cycle typically spans across 4–5 s. In the context of respiration research, even the long time course of mental imagery could be accommodated.

#### Attention

Some studies have used simultaneous multiple clock stimuli to investigate attention (Carlson et al., 2006; Hogendoorn et al., 2007; Keetels & Vroomen, 2011). For example, Carlson et al. (2006) used an array of clock stimuli to investigate the speed of visual attentional shifts. In their study, participants were required to shift their attention from the centre of the screen to one of ten clocks arranged in a circle and to then give a time report of the chosen clock. The would-be reported clock pointer was indicated via a visual cue at the start of the trial. Carlson et al. investigated how the type of cue (peripheral vs. central cues) would affect time reports given by the participant. Posner (1980) posited a distinction between two types of cues that motivate visual attentional shifts: peripheral cues, which are presented at a target location (e.g., at the right side of the screen), and central cues, which are presented at the central place of fixation and symbolically represent the cued location (e.g., via a directional arrow). Peripheral cues have been claimed to draw attention automatically whereas central cues require volition (Jonides, 1981). Central cues require its meaning to be deciphered and so have consistently been found to result in slower attentional shifts relative to peripheral cues (Müller & Rabbitt, 1989). Carlson et al. (2006) found that the time report of the attention clock (referenced by the cue onset) was earlier for peripheral cues than for central cues, which is in line with previous studies reporting faster processing of peripheral than central cueing (Jonides, 1981; Müller & Rabbitt, 1989).

### The mechanisms involved in the complication clock method

Here, we detail the numerous mechanisms involved in the formation of a time report. As discussed in the *Introduction*, the potential biases and validity of time reports have been debated mainly because of two fundamental presumptions involved with the complication clock. Here, we address the first. The pointer and its uniform motion, as representative of the unfolding of time, allows the participant to decide when they think a conscious event occurred. However, due to the fact that participants are localising the position of the moving pointer, which serves as a medium for time, there are a number of biases involved in the judgement. We include not only those that have been directly discussed under the umbrella of the complication clock, but also peripheral mechanisms that may be implicated in the clock method. Despite the apparent simplicity of forming a time report, our introduction to the complication clock showed that a single time report is formed by numerous mechanisms and biases. Though any single use of the complication clock may not involve all the mechanisms discussed here, there is no doubt that they involve a combination of them, depending on the task demands of the experiment. Before proceeding with the mechanisms, we briefly introduce the bias and variability in the results produced by the method.

#### Bias and variability in the complication clock

The ‘eye and ear’ method – which served as the inspiration for the complication clock developed by Wundt – was used during the eighteenth and nineteenth centuries to time stellar transits (Cairney, 1975; Sheehan, 2013). Astronomers noted the time when the star being observed appeared in their telescope field then noted the time before and after the star crossed a central line on the telescope.

In 1796, David Kinnebrook, an assistant at the Greenwich Royal Observatory, lost his job because his ‘eye and ear’ observations did not match that of his supervisor, the Astronomer Royal Nevil Maskelyne (Hoffmann, 2007; Sheehan, 2013). Friedrich Wilhelm Bessel (1824), an astronomer from Prussia, would later show that the ‘eye and ear’ method gave rise to marked individual differences, which he referred to as an ‘*involuntary constant difference*’ or ‘*personal equation*’, arguing that individual observers would produce uniquely biased results in their measurements regardless of their level of training. The complication clock method shares core features with the ‘eye and ear’ method (e.g., moving reference, transient event, and fused judgement). It of course is not immune to biases in the results produced.

No matter the research question, all participants must watch a rotating pointer and report the position of the pointer at the time the event of interest occurs. In the case of externally generated events, a report error is always calculated as the difference between the time report (i.e., the reported pointer position) and the actual position of the pointer at the event occurrence for further processing. If participants can report the event timing without any bias via the complication clock method, the report error should in theory have an average value of 0. However, this is far from the reality.

For example, when reporting the onset time of a sound, the average report errors range from −40 ms to 33 ms among participants in a study by Miller et al. (2010). This not only illustrates a bias in the time report but also a variability of the bias among participants in the same study. In addition, the report errors from individual trials of the same participant are also highly variable (e.g., with an average standard deviation for about 111 ms in Miller et al., 2010). Across different studies, the group average report error of the onset of a sound can range from a negative error average of −166 ms (Danquah et al., 2008) to a positive error average of 95 ms (Wolpe et al., 2013). In the case of internal events, such as decision times for keypresses, the results of Miller et al. (2010) provide clear evidence that people cannot give accurate time reports with the clock method. In their study, a substantial number of the decision times reported were implausibly early or late. For example, 10–15% of decision times were reported to have been made after the keypress had already occurred.

#### Mechanisms related to time reports

**Fig. 3:**
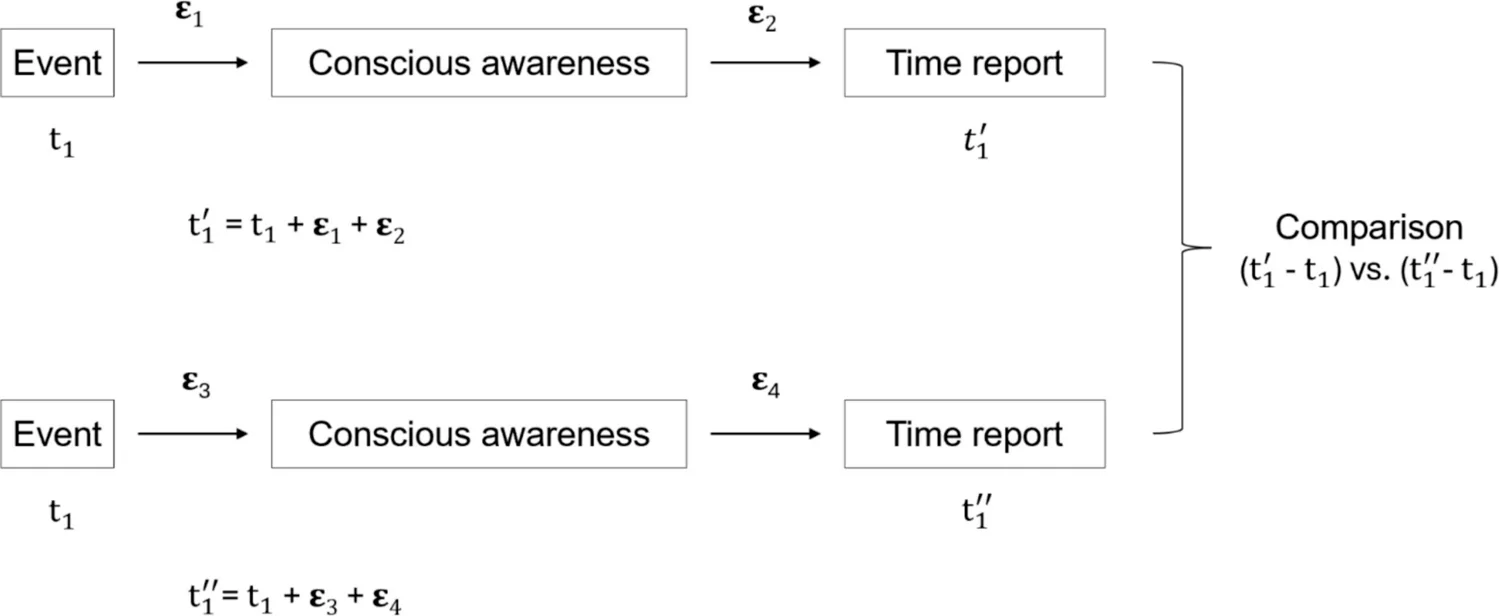
Schematic representation of the formation process of time reports with the complication clock.** Upper row:** An event occurs at t₁; the interval ${{\boldsymbol{\upvarepsilon}}}_{1}$ (always positive) denotes the latency from event onset to conscious awareness; ${{\boldsymbol{\upvarepsilon}}}_{2}$ denotes the net effect of biasing factors on the time report (which can be positive or negative). The resulting time report is ${\mathrm{t}}_{1}^{^{\prime}}={\mathrm{t}}_{1}+{{\boldsymbol{\upvarepsilon}}}_{1}+{{\boldsymbol{\upvarepsilon}}}_{2}$. Because ${{\boldsymbol{\upvarepsilon}}}_{2}$ can be negative, ${\mathrm{t}}_{1}^{^{\prime}}$ can fall before ${\mathrm{t}}_{1}$. **Lower row:** A second condition for the same event, with latency ${{\boldsymbol{\upvarepsilon}}}_{3}$ and bias term ${{\boldsymbol{\upvarepsilon}}}_{4}$, yielding ${\mathrm{t}}^{{^\prime}{^\prime}}_{1}{\mathrm{t}}_{1}+{{\boldsymbol{\upvarepsilon}}}_{3}+{{\boldsymbol{\upvarepsilon}}}_{4}$. **Right:** The between-condition comparison (${\mathrm{t}}_{1}^{^{\prime}}-{\mathrm{t}}_{1}$) vs. (${\mathrm{t}}^{{^\prime}{^\prime}}_{1}-\mathrm{t}_{1}$). A difference between these two report errors is typically attributed to a difference in time perception. As we analysed, the difference may equally arise from differences in conscious-awareness latency ($\boldsymbol\upvarepsilon_{1}$ vs. ${\boldsymbol\upvarepsilon}_{3}$), from differences in biasing factors ($\boldsymbol\upvarepsilon_{2}$ vs. $\boldsymbol\upvarepsilon_{4}$), or from both. Biasing factors in $\boldsymbol\upvarepsilon_{2}$ and $\boldsymbol\upvarepsilon_{4}$ are not restricted to post-awareness processes. Attentional allocation before event onset can shift the time report independently of the awareness latency (cf. Wang et al., 2025)

To report the timing of an event using the complication clock, the event must first be consciously registered. Conscious awareness takes time, and this latency enters the report before any further bias operates. The reported time is therefore the sum of the actual event time, the latency to conscious awareness, and the net effect of all systematic biases operating between event onset and the formation of the report (Fig. 3). Some studies attempt to circumvent the bias problem through between-condition comparisons (e.g., comparing time reports for an identical sound that is triggered either by a keypress or by the computer). The rationale is that if all biases are equal across conditions, any difference in time reports reflects the experimental manipulation alone. As we analyse below, however, this equal-bias assumption may not hold.

#### The event

The event timing itself is sometimes ambiguous, as events do not occupy one discrete moment. For example, acoustic events can span across time, and the moment that one perceptually registers its occurrence, known as the perceptual centre – or p-centre – of a stimulus (Morton et al., 1976; Scott, 1998), can differ from its temporal onset based on the acoustic properties of the event. Pockett and Miller (2007) showed that keypress time reports changed depending on whether participants were told to report the beginning or completion of their keypress movement. However, most studies do not specify which point of the keypress to base time reports on, leaving space for between-participant variability for instruction interpretation and thus the possibility of systematic differences in time reports that are independent of experimental manipulations. The influence of p-centre is perhaps even more pronounced in studies that do not use simple stimuli like brief sounds, and attempt to compare time reports for sounds of different emotional valences (Barlas & Obhi, 2014; Yoshie & Haggard, 2013). However, it should be noted that Yoshie and Haggard (2013) attempted to control for p-centres for the stimuli in their study, meaning that, in principle, the effect of p-centres could be controlled for in experiments.

#### The latency of conscious awareness

Although rarely discussed nowadays, a key topic of debate at the time of the Libet studies was the timing of conscious experience. In fact, Wundt argued that allocating attention to a particular perceptual stream would lead to earlier time reports to events of that stream (known as *prior entry*). The key task of the complication clock experiment is that of the participant reporting the timing (via spatial localisation) of their *conscious* experience of an event. Therefore, a crucial element to be discussed is that of the latency of conscious experiences (e.g., the experience of hearing a tone or seeing a visual stimulus). Participants, after all, are not reporting the timing of when an event objectively occurs, but rather their conscious experience of the event. Although the two tend to be conflated, there was fierce debate about the latency of conscious experience from the publishing of Libet’s studies (Libet et al., 1964, 1979) up until the early 2000s. Recall that the early studies by Libet and colleagues led to the development of *backwards referral*, wherein the timing of a conscious sensation is back-dated to the time of the onset of the stimulus. This idea was highly controversial (Churchland, 1981; Gomes, 1998, 2002a; Pockett, 2002; van de Grind, 2002), but the discussion surrounding this idea also serves to elucidate how the concept of the latencies surrounding our conscious experience is no simple matter.

Libet based his notion of *backwards referral* on his finding that it took approximately 500 ms of direct cortical stimulation to produce a conscious sensation. Against this interpretation, Gomes (1998) put forward the distinction between *real latency* and *experimental latency*. The example Gomes used was a neural structure wherein a train of electrical pulses totalling 200 ms was applied so that a muscular contraction appeared 50 ms after the last electrical pulse. If, instead, the train of pulses lasted for 500 ms, the contraction response would last for 300 ms, beginning 250 ms after the onset of the first pulse. The 250-ms period between the onset of the pulse and the first muscular contraction would be the *experimental latency*. However, in this example, the *real latency* would be 50 ms, with the first 200 ms being the preparatory period to build the neural system to its necessary excitatory state. Gomes argued that Libet’s interpretation of his experimental results ignored this distinction and instead conflated the two types of latencies associated with producing a conscious sensation.

van de Grind (2002) recognised that the issue of the timing and latency of conscious experience was essentially about the relation between physical timing, neural timing, and experiential (mental) timing, coining the issue “Libet’s problem”. According to van de Grind, while Libet put forward the notion of *backwards referral* to explain his experimental findings, his opponents sought to explain the findings on the intervening neural processes that translate the physical timing of stimuli to experiential timing. For example, for different types of conscious experiences (such as stimulation of the skin or hearing a sound), there are presumably different latencies involved from first reception of the stimulus and the preceding (nonconscious) neural processes associated with producing the corresponding conscious experience.

Therefore, to judge the reliability of the complication clock, one must be aware of how the latencies of physical events may translate to conscious experience, which participants base their time reports on. When participants are instructed to report the timing of a conscious experience, they often correspond to events of different sensory modalities such as a moving pointer versus a brief tone. Furthermore, in this same example, the two stimuli are different in that one is a tonic stimulus while another is transient, with the possibility that the latencies involved are different depending on the nature of the stimuli (Gomes, 1998).

For example, Libet et al. (1983) had sought to use time reports for brief stimulation applied to the skin as a method to control for potential errors when participants gave their timing reports of intention. The idea being that if timing reports for skin stimulation resulted in negative errors, then that error value could be used to correct the timing reports for intention timing reports, revealing the ‘true’ intention timing. However, this only holds if the latencies for conscious awareness of intention and skin stimulation are the same. Furthermore, since they are both tied to the spatial localisation of the pointer (a stimulus that is not transient but moves throughout the duration of the trial), the temporal relation between the two stimuli and the moving pointer also needs to be considered. As Rollman (1985) argued, there is likely also a latency involved with becoming conscious of the position of the pointer; the latency of the continuous, conscious experience of the pointer is likely different from the latency of a momentaneous event.

An oft-discussed phenomenon when discussing the results of the Libet studies using the complication clock is the flash-lag effect (Nijhawan, 2002; Whitney & Murakami, 1998; Yook et al., 2024). When a flashed object is presented at the same location as a moving object, rather than appear at the same location, the flashed object is perceived to be behind the moving object. Whitney and Murakami (1998) proposed that perception of the flashed object lagging behind the moving object was because the latency of the moving object was shorter due to prior facilitation of motion-sensitive receptor cells along the path of the motion. This is highly relevant for the complication clock task as a discrete event (such as a visual flash or a sound) is often introduced while participants are already observing the movement of the pointer. If it is indeed true that latencies are shorter for moving objects, then this could introduce a systematic bias in the complication clock task. However, it should be noted that most flash-lag experiments usually only involve visual stimuli, and this phenomenon is perhaps less relevant for studies that use auditory events. Danquah et al. (2008) directly investigated the timing of different sensory modalities by having participants give time reports in the complication clock with stimuli of different sensory modalities. They found that tactile events lead to later time reports compared to auditory and visual events.

Studies using the simultaneity task are also relevant to consider for latencies. In this task, participants have to judge whether a pair of presented stimuli appear simultaneously (Zampini et al., 2003). Zampini et al. (2003) conducted several experiments measuring audiovisual temporal order judgements. One important finding was that the visual stimulus had to precede the auditory stimulus by about 60–80 ms in order for the two stimuli to be perceived as simultaneous. They argued that the reason for this discrepancy is that neural transduction latencies at the peripheral sensory epithelia were different for the two sensory modalities. Compared to the time it takes for visual stimuli to be transduced at the retina, it takes much less time for auditory stimuli to be transduced (King & Palmer, 1985; Spence & Squire, 2003). This may have implications for the complication clock task as stimulus presentations, often in the form of an auditory event, are paired with judgements of the pointer, which is a visual stimulus (however, it should again be noted that the pointer is a tonic stimulus vs. events which are often transitory in nature). The idea of the ‘personal equation’ (Bessel, 1824) is also relevant here, as Grabot and Van Wassenhove (2017) have demonstrated stable interindividual differences in the perception of temporal order across and within sensory modalities.

#### The time report with the complication clock

It has been long known that clock parameters can influence time reports. Wundt’s early experiments (1873) investigated the effect of pointer speeds on time reports. He found that positive errors became more likely as the pointer speed increased. The effects of pointer speed were also replicated by his contemporaries (Burrow, 1909; Geiger, 1903; Pflaum, 1900; von Tchisch, 1885). Geiger’s (1903) complication experiments revealed patterns of displacement that were dependent on the position of the pointer when the auditory stimulus was presented. When the sound was presented while the pointer was travelling clockwise in a downward direction, participants were more likely to give a positively biased time report. However, when the sound was presented while the pointer was on the left side of the clock face (i.e., travelling in an upwards direction), participants were more likely to give negatively biased reports.

The positive bias associated with the downwards motion resembles the representational gravity illusion (Hubbard, 2019), an illusion in which the judged location of a moving object is displaced in the direction of implied gravitational attraction. The representational gravity illusion joins another spatial illusion known as representational momentum, in which the memory of the location of a recently viewed object is mistakenly displaced forward in the direction of the object motion (Hayes & Freyd, 2002; Hubbard, 2005). In their study, Joordens et al. (2002) reported that participants tended to have a positive bias with their time reports, which was attributed to the representational momentum phenomenon. However, these results are partially in conflict with the fact that the time report error of a stimulus can also be negative in some studies (Cao, 2024; Haggard et al., 2002). This reflects the fact that localising the pointer may involve several biases in conjunction with the task demands of the experiment itself.

Recent work has extensively looked at the effect of physical characteristics of the complication clock on time reports (Ivanof, Terhune, Coyle, & Moore, 2022a, Ivanof, Terhune, Coyle, Gottero, & Moore, 2022b). We summarised these results in Table 1. The fact that stimulus features of the complication clock can significantly affect time reports makes it difficult to compare the results between studies, as experiments differ not just between research questions but also between laboratory protocols.

**Table 1:** List of studies investigating physical parameters of the clock stimulus and their results

Study	Subject area	Parameter investigated	Results of parameter investigation
Wundt (1873)^a^	Sensory processing – complication task	Pointer speed	Increased pointer speeds led to a higher chance of positive biases; slower speeds led to negative biases
Geiger (1903)	Sensory processing – complication task	Pointer position during tone onset	Tones presented on the right side of clock led to positive biases; tones presented on left side led to negative biases
Pockett & Miller (2007)	Libet task (time *M* – keypress time)	Trigger for movement^b^ Report start/end of movement^c^ Spot luminance^d^ Clock radius Gaze behaviour^e^ Spot size Spot speed	No effect of urge/decision on *M* judgements Participant judgements were systematically earlier when told to report start of movement compared to end of movement No effect of spot luminance on *M* judgements No effect of clock radius on *M* judgements No effect of gaze behaviour on *M* judgements No effect of spot size on *M* judgements No effect of spot speed on *M* judgements
Danquah et al. (2008)^f^	Libet task (time *S* – time of electric pulse tactile delivery)	Pointer speed	*S* judgements were systematically later as pointer speed increased and became closer to true stimulus delivery time
Ivanof, Terhune, Coyle, & Moore (2022b)	Libet task (time *W* – intention awareness)	Pointer speed Clock face markings Pointer length Clock radius	Greater speeds led to smaller gap between *W* and actual keypress time Minimal (but not no) clock face markings led to smaller gap between *W* and actual keypress time Pointer lengths did not have an effect on *W* judgements Clock radii did not have an effect on *W* judgements
Ivanof, Terhune, Coyle, Gottero, & Moore (2022a)	Temporal binding	Pointer speed Clock face markings Pointer length	Greater speeds led to larger outcome binding, but not action binding Different clock face markings did not have an effect on temporal binding Pointer lengths did not have an effect on temporal binding

^a^ Results by Wundt (1873) were replicated by von Tchisch (1885) and Pflaum (1900)

^b^ Participants were instructed to either make a definite decision or spontaneously experience an urge before performing the keypress

^c^ Participants were instructed to base their *M* judgements on either the initiation of the keypress or the conclusion of the keypress movement

^d^ A rotating spot was used instead of a pointer

^e^ Participants were instructed to either fixate centrally or follow the rotating spot during the course of the trial

^f^ Danquah et al. (2008) also investigated the effect of different sensory modalities of event presentation (auditory, visual, and tactile events). They found no difference between auditory and visual modalities but tactile events led to later time reports compared to the other two modalities

Another important consideration for time reports is eye-movement behaviour. Because the complication task is a visuospatial task, eye-movement behaviour plays an important role in pointer localisation. The role of eye-movement behaviour was also recognised in the early complication experiments (Angell & Pierce, 1892; Burrow, 1909; Dunlap, 1910; Geiger, 1903). Angell and Pierce (1892), for example, suggested that positive errors were due to the eyes briefly following the pointer after the stimulus presentation. When the pointer was slow, they argued that negative errors were due to participants moving ahead of the pointer, then overcorrecting when they heard the sound.

However, it should be noted that early work using the complication clock involved a much larger clock apparatus (in some cases, a pendulum was used instead of a clock). Participants were unable to fixate centrally and had to actively track the moving pointer. Furthermore, although different eye-movement behaviours such as fixation versus smooth pursuit can have a significant effect on the ability to localise moving objects (Van Beers et al., 2001), Pockett and Miller (2007) found no effect of gaze behaviour on pointer localisation.

In addition, the way the participants approach the experimental task may also have an effect on time reports. The strategy by which participants approach an experiment is often assumed to be fixed a priori (Szollosi & Newell, 2020). However, even for a task as simple as the complication task, participants – as a result of their different dispositions and representations of the task – can have different strategies to localise pointer positions. In the complication experiments, Geiger (1903) broadly identified two types of participants, which he designated as ‘naive’ or ‘reflecting’. ‘Naive’ participants tended to approach the task in a more nonchalant manner while ‘reflecting’ participants tended to be more vigilant. He found that ‘naive’ participants were more likely to make negative report errors whereas ‘reflecting’ participants were more likely to commit positive report errors. The substantial individual variability observed in time reports (Miller et al., 2010) may be indicative of different strategies participants use to localise the pointer as well as changes to participant representations of the task throughout the duration of the experiment.

#### Between-condition comparison of time reports

One potential solution to the bias problem in the complication clock is to compare the time reports for the same event across different conditions. The idea is that the biasing factors are the same between an experimental condition and a baseline condition. Therefore, any difference in the time report would reflect an effect of the experimental manipulation. For example, in temporal binding, time reports of sounds are compared between when the sound is triggered by the participant’s keypress and when it is triggered by the computer. However, recent advances show that the assumption of an equal bias between conditions may not hold.

The cue-integration account of temporal binding posits that time reports of an event are contributed by cues from different sources (Cao et al., 2020; Klaffehn et al., 2021; Wolpe et al., 2013), which can be captured by a Bayesian model (Legaspi & Toyoizumi, 2019). For example, the time reports of a sound have an additional cue in the instrumental condition (i.e., a keypress) compared to the baseline condition. Since the additional cue occurs before the event to be reported, the result of integration is an earlier reported time (i.e., outcome binding). Therefore, according to the cue-integration account, temporal binding results from a change in the cognitive representation of the event.

Recent work using the clock has also looked at eye movements (Huang et al., 2022; Hunt & Cavanagh, 2009; Kosovicheva & Bex, 2020). Huang et al. (2022) investigated the role of eye movements in outcome binding. They found that, in the 250-ms delay period leading up to the sound, there were less saccades directed towards the pointer in the instrumental outcome condition compared to the baseline outcome condition. The lack of these saccades in the instrumental outcome condition meant that participants were more likely to localise the pointer closer to its position at the time of keypress.

The key implication here is that some of the variability in results between individuals and conditions may partially be explained by differences in eye-movement behaviour. Different experimental conditions might require different types of eye-movement patterns from participants, which may also be linked with differences in between-condition task demands such as visuospatial attention. As such, some results of the studies that have used the complication clock may be partially explained by between-condition differences in eye-movement behaviour and may not necessarily reflect the experimental manipulations the researchers were aiming for.

Related to eye movements is the role of attention in time reports (Cao 2024; Chen et al., 2025; Haggard et al., 2002; Haggard & Cole, 2007; Schwarz & Weller, 2023; Wang et al., 2025; Yabe & Goodale, 2015). Distinct mechanisms have been raised under the umbrella of attention. Attention has been proposed as a general cognitive resource to influence time reports, since reporting the pointer position requires the deployment of cognitive resources. For example, in the context of temporal binding, Haggard and Cole (2007) showed that if participants did not know which event (the keypress or the keypress-triggered sound) was required for the time report until the keypress had been performed, the reported keypress time and sound time almost collapsed to the same point somewhere between the keypress and the sound. However, if participants knew which event to report beforehand (i.e., attention could be focused on the event in question), the time report was much less biased.

One recent study investigated the role of visuospatial attention in time reports (Cao, 2024). Given the fast-rotating speed of the pointer, it is a challenging task to precisely locate the pointer at a specific time point, which is also a key factor leading to the variability in time reports across trials. Therefore, the distribution of visuospatial attention around the clock face at time points close to the event of interest may be a highly salient cue for participants to locate the pointer (i.e., making the time report). Indeed, it was shown that the distribution of visuospatial attention could predict the time report in a single condition, and that the difference in the distribution of visuospatial attention between two conditions could predict the difference of time reports between the two conditions, even when eye movements were controlled for. Furthermore, experimentally manipulating the distribution of visuospatial attention led to predictable changes in time reports, thus corroborating the functional role of attention to pointer localisation (or time reports) rather than the other way around (Chen et al., 2025).

### The philosophy of time presupposed by the complication clock

In this section, we discuss the second fundamental presumption of the complication clock. Throughout this article, it has been claimed that the complication clock assumes an isomorphism between time perception and the spatial form of the clock. Beyond the practical implications detailed in the previous section such as biases of pointer localisation, there are also theoretical concerns. The complication clock transforms a spatial array into an objective measurement of subjective time. The form of the complication clock assumes an isomorphism between the spatial array of the clock – which is discrete and linear – and the psychological experience of time,^3^ When we say that the spatial form of the clock is discrete, this is because time reports given by the participants are of a discrete nature. Therefore, it presupposes a form of time perception that is also discretisable and linear, as the perception of *when* an event occurs directly corresponds to its judged spatial position on the clock face.

The equivalence between time and space is intuitive because time has often been tightly linked to the concept of space (Núñez & Cooperrider, 2013; Sima & Sanayei, 2024). For example, we often speak of time metaphorically in terms of space itself (e.g., ‘*a long time*’ or ‘*time flies*’). However, recent work has sought to challenge this view of time perception (Guo et al., 2025; Robbe, 2023; Salet et al., 2022). For example, Salet et al. (2022) argued against seeing time perception as containing stopwatch-like intervals and for an intrinsic-adaptive view of timing, where peoples’ experience of time is essentially adaptive to intervals and regularities in the natural world. Robbe (2023), based on the work of philosopher Henri Bergson, argues that because we have memory, the contents of our inner life qualitatively evolve and permeate into one another, with the consequence that our experience of time is indivisible and unable to be quantified, unlike space. Interestingly, Wundt proposed something similar by arguing that our perception was shaped not only by sensations but by the continuous merging of memories and attention (Wundt, 1873). There is some empirical support for these claims. For example, it has been shown that working memory can directly alter perception (Teng & Kravitz, 2019). In their study, Teng and Kravitz (2019) found that holding an object in mind directly affected the perception of new stimuli via a shift in psychophysical discrimination thresholds. Harrison and Tong (2009) found that information in visual working memory is maintained in early cortical areas. Thus, the intertwining between past experience, memory, and ongoing perception would suggest that a clear demarcation between moments in our temporal experience is conceptually difficult.

**Fig. 4:**
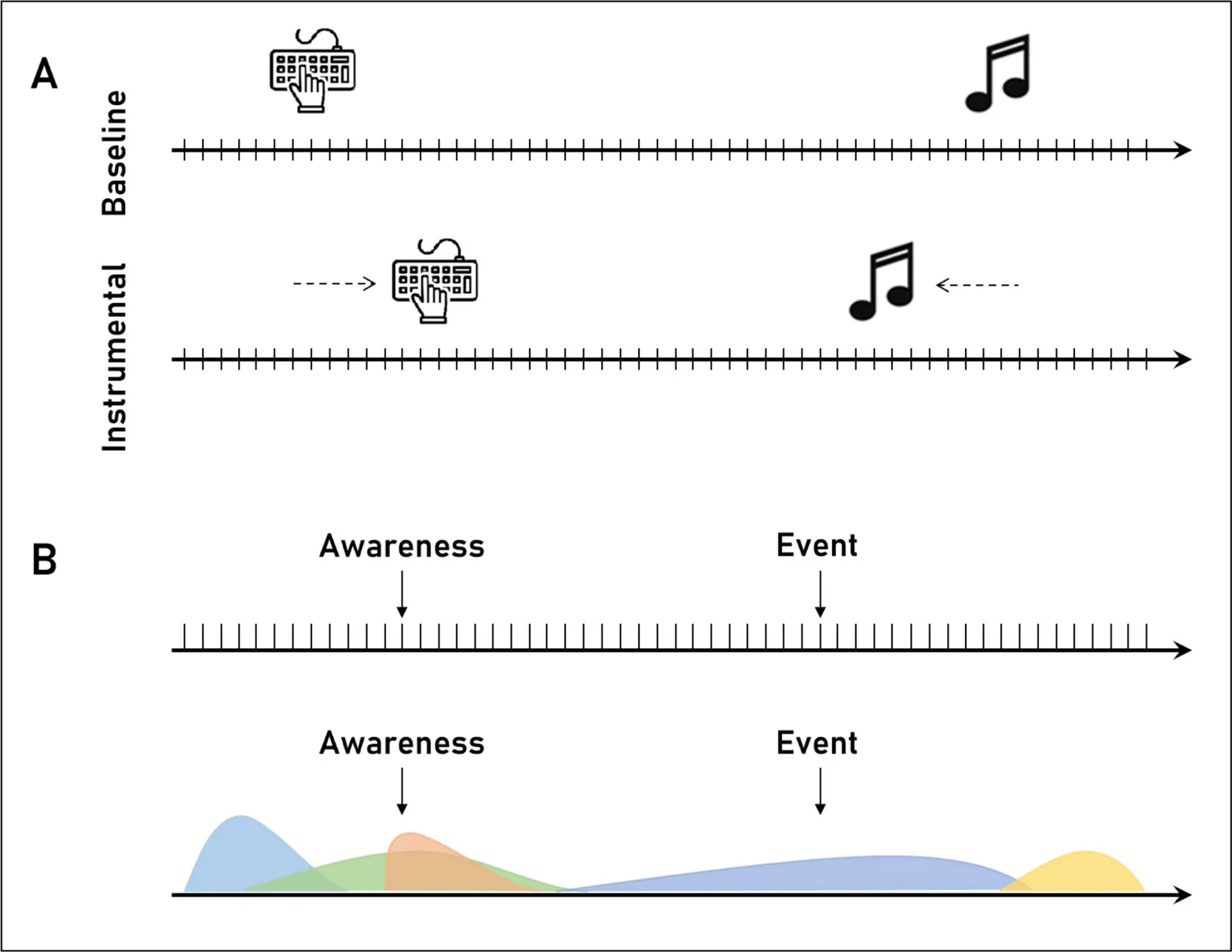
Implications of the space-time isomorphism assumption. The complication clock is a method that maps time to space and so assumes an isomorphism between the spatial array of the clock form – which is discrete and linear – and our subjective experience of time. This is an issue that implicates our experience of *duration*. **(A)** One example is temporal binding, where the phenomenal duration between action-effects is ostensibly shortened. In action binding, the mechanisms that would allow for an action to be perceived as occurring later than it had actually occurred (and vice versa for outcome binding) are not clear. However, this phenomenon can become more intelligible if we substitute the spatial representation of time of the clock into our psychological experience of time without identifying a clear mechanism of how it may arise. (**B**) Discrete versus continuous temporal experience. *Upper timeline:* The clock’s spatial array represents experience as a sequence of discrete, linearly ordered points. *Lower timeline:* An alternative conception in which cognitive and affective states evolve continuously, overlap, and permeate one another (illustrated by overlapping distributions). The discrete-linear format of the clock favours interpretations in which internal events (e.g., intention or affect onset) occupy identifiable time points. However, it is less suited to represent processes in which the boundary between states is gradual or context-dependent (Banks & Isham, 2009; Lai et al., 2012; Miller et al., 2010)

One of the concerns of the critique of the traditional view of time perception is with our experience of *duration* or with the ongoing experience of time. Although the clock is primarily concerned with the discrete moment of *when* an event psychologically occurs, duration can sometimes be *implied* by the results of the experiment. For example, temporal binding posits that when action-effects are coupled, the subjective experience of our action is perceived as slightly later than when it actually occurred, and that the outcome is perceived as slightly earlier. That is, the perceived duration between the two is shortened. In the case of action binding, it is unclear how our present, phenomenological experience of an action that is performed in the here and now can be *perceived* as occurring later than it actually is. However, such an issue can be sidestepped and made more easily intelligible if we assume that the spatial representation of time can be transmuted into our psychological experience of time. For example, when depicting the temporal binding phenomenon, it is typically shown as events being drawn towards one another in space (see Fig. 4). For action binding, the action is represented later in space, so it is assumed that the same thing is happening with our experience of time. What may be happening, as Gibson (1979) argued, is that we assume our experience of time to be like an empty receptable that is then filled up with events. Process-oriented approaches to time perception (Robbe, 2023; Salet et al., 2022), however, would claim that our experience of passing time can never be truly repeated as our experience of time does not occur in such a discrete, linear manner. This can pose a problem for effects like temporal binding which tries to make the shortening of duration a general phenomenon associated with action-outcome sequences.

The second issue concerns the measurement of internal phenomena such as volition or mental imagery. In the complication task, a discrete pointer position is chosen, but this can hide the fact that it is not exactly clear when internal phenomena begin and end. By being able to discretely and spatially represent *when* internal phenomena occur, one can be biased into thinking that our complex inner life underpinning behaviour can be simplified into discrete, linear arcs (see Fig. 4; Gomez-Marin & Ghazanfar, 2019; Nicholson & Dupré, 2018). An example here is between two competing interpretations of the readiness potential (Libet et al., 1982; Schurger et al., 2012). These two competing interpretations also illustrate different approaches to how the complication clock can be used. In the classical interpretation of the readiness potential, which is mostly associated with the Libet experiments (Libet et al., 1982), volitional action follows linear, discrete steps: there is an unconscious neural decision to initiate movement corresponding to an accumulation of neural activity. This process is directed specifically towards the production of movement, with accumulation of neural activity ceasing after movement commences. This linear interpretation of volitional action is contrasted to the late-decision account described in Schurger et al. (2021). In this account, volitional action does not follow linear, discrete steps but instead is a dynamic process shaped by participant goals as well as constraints of the environment and task.

The two competing interpretations of the readiness potential also illustrate two different approaches to using a tool like the complication clock. In the traditional interpretation, participant *intention* is localised on the clock as occurring earlier than the action (however, it has been shown that such judgements are highly variable as demonstrated by Miller et al., 2010), and neural activity occurs earlier than the identified awareness of intention time. It was therefore argued that these stages follow one after another in a step-by-step fashion. In this way, this interpretation of volitional action as a result of discrete steps also follows the spatialised representation of time as the complication clock. On the other hand, Schurger et al. (2012) use the complication clock in an entirely different manner to investigate the late-decision interpretation of the readiness potential. Here, participants are not assessed on their awareness of intention or keypress onset. The clock instead serves as a visual representation of elapsed time. The representation of increasing elapsed time can instead serve as a method to manipulate urgency and the motivational state of the participant.

Considering the shift from primarily investigating external events to more internal phenomena, the issues discussed here are perhaps more relevant in the modern usage of the complication clock. For example, the transformation of space into time via pointer localisation allowed for Franikowski et al. (2021) to conclude that cognition always preceded affect and for these two stages to operate in a serialised manner. However, it has also been argued that the primacy of cognition versus affect are contextually dependent (Lai et al., 2012). The discrete, spatialised nature of time assumed by the clock form can make it difficult to study internal phenomena that may potentially be more dynamic in nature, thereby biasing interpretations of cognitive phenomena.

### Addressing the practical and theoretical issues of the complication clock

Throughout its history, despite the debate surrounding its accuracy, the complication clock has been treated as essentially a passive measurement device that more or less could accurately capture the timing of discrete mental events. Reddy (2022) correctly claimed that the complication clock was essentially a motion-localisation task. Here, we address how one can use the complication clock whilst addressing the practical and theoretical issues discussed above. When investigating simple external events such as in sensory-processing studies, one perhaps need only to consider how to mitigate potential biases such as spatial attention or eye movements. However, modern studies of the complication clock deal with phenomena such as volition and sense of agency with profound implications for human cognition.

#### Key presumptions

As first discussed in the *Introduction*, there are two key presumptions to the complication clock. They are both related to the spatial form of the complication clock. Firstly, the pointer and its uniform motion, as representative of the ongoing flow of time, allows the participant to decide when they think a conscious event occurred. However, it is the same movement of the pointer that is the main cause of the different biases that affect participant time reports. For example, because participants are tracking the pointer in addition to paying attention to the to-be-presented or internal event, there will inevitably be variations in attention as well as eye movements. Indeed, eye movements can be affected even for internally directed actions such as mental imagery (Ceh et al., 2020). As previously discussed, latencies for a moving object like the pointer may also be different from those of a discrete event such as a tone or visual flash, potentially affecting the conscious experience on which time reports are based. This means that the complication clock cannot be considered to be merely a passive instrument to measure the timing of events, but is an active element that the participant interacts with over the course of the experiment. Secondly, the complication clock assumes an isomorphism between the spatial array of the clock form and our psychological experience of time. Because the time reports given by participants via the pointer are discrete, it also implies the same property to our ongoing mental processes. However, owing to both theoretical (Robbe, 2023; Salet et al., 2022; Wundt, 1873) and empirical reasons (Harrison & Tong, 2009; Teng & Kravitz, 2019), we argue that such discretisation of our mental experience of time is problematic.

#### Implications

The two presumptions have important implications for how future studies should consider using the complication clock. The implications can be divided based on the theoretical orientation of the research area using the clock and affect how the study using the clock should account for potential biases and theoretical issues. We briefly discuss the implications the key presumptions will have on the different types of studies using the complication clock, then discuss potential solutions.

#### Studies investigating basic sensory processing need only to focus on the first key presumption

Studies in sensory processing (e.g., Sanford, 1971, 1974; Seifried et al., 2010) will mainly need to consider how to address potential biases in time reports. This is because time reports in these studies rarely have far-reaching implications regarding the temporal properties of our cognitive machinery and are mainly concerned with, for example, the speed of processing stimuli. For example, Seifried et al. (2010) investigated how temporal preparation affected the speed of processing a target tone by using time reports. In their study, participants gave earlier time reports to target tones when the time between the warning tone and target tone was short and vice versa, suggesting that with proper temporal preparation, participants were able to process the target tone earlier. The implications for this study mainly refer to how temporal preparation and predictability affect our ability to create time reports. In such studies, the main concern would be to control for between-condition differences in biases that could systematically affect time reports. Although most modern studies using the complication clock have moved beyond investigating sensory processing, there also exist recent studies within the sensory processing tradition that use time reports to investigate the timing of aversive stimuli (Castellote & Valls-Solé, 2019; Sanegre et al., 2004; Valls-Solé et al., 2012). These studies, like the early ones using the clock, primarily only need to consider how to address potential biases in time reports.

#### Studies concerning the temporality of cognition should align the clock form with the theoretical construct

There have been several discussions in this review about how the spatial form of the complication clock assumes a form of time perception that is also linear and discrete. This form also implies a type of seriality for cognitive processes, such as in the case of intention and volition in the Libet studies. Therefore, when using the complication clock, one should consider how their theoretical implications yielded by the experiment may be potentially constrained. This is the main difference between studies that investigate the temporal properties of cognition and those that mainly focus on sensory processing. However, both types of studies using the complication clock need to keep in mind the potential biases shaping time reports.

#### Potential solutions

Here, we discuss several solutions to the problems, both empirical and theoretical, to using the complication clock discussed through the review. We first list several solutions to the mechanistic issues of the pointer movement such as eye movements or between-condition differences in attention. We then discuss the *affordance competition hypothesis* (Cisek & Kalaska, 2010), a novel interpretation of action control that can elucidate how the complication clock could be adapted towards theories that argue for parallel processes in cognition as opposed to serial processes.

#### Temporal resolution

An important consideration when designing a study using the complication clock is the temporal resolution of the time report. Different research questions require different temporal resolutions regarding time reports for events. For example, using the complication clock, Park et al. (2022) showed that mental imagery was more likely to occur during the expiration phase of the respiratory cycle. It may be perfectly feasible to investigate the timing of events such as sounds or even perhaps motor intention during the respiratory cycle as respiratory cycles typically last about 4–5 s.

#### Identification of biases

Time reports are the result of not only the event-in-question but the net effect of all biasing factors. The task demands of the complication clock task will determine the combination of different biases affecting the time report. It may be difficult, if not impossible, to ascertain exactly which biases are at play for a single experiment. This is particularly an issue in studies that compare the timing of the same event. Any difference in the report error between conditions is usually attributed to the factor being manipulated. This line of reasoning assumes impacting biases to remain the same between conditions, whereas the difference in report errors may simply reflect a difference in the impact of biasing factors. A notable example is the between-condition differences in visuospatial attention in temporal binding (Cao, 2024; Wang et al., 2025).

Due to the difficulty in identifying all possible biasing factors, the best outcome here may be to control for the most obvious biases in an experiment. For example, an eye control protocol could be implemented to limit the impact of eye movements. Simultaneously, one should make sure that the research question can tolerate the biases of report errors and is not reliant on time reports having precise temporal resolution.

#### Biases for external versus internal events

The impact of biases for external events can be varied and estimated as the actual timing of the event is known. However, the bias of internal events cannot be easily estimated. This is because the timing properties of internal events (e.g., the onset, the duration) are not known. Therefore, one can only make a reasonable guess as to whether the time report corresponds to the internal event in question. This is also related to the issue of temporal resolution. If one requires precision in the time report of an internal event, as the timing properties of internal events are unknown, it may be difficult to tell if obtained time reports merely reflect biases or the actual event in question. For example, the obtained time report may truly capture a distinct internal event or may simply be the result of various biases such as visuospatial attention (Cao, 2024; Wang et al., 2025). Therefore, if the goal is to measure the timing of internal events, one ought to be able to accommodate the coarse temporal resolution of time reports in the complication clock.

In reality, all three issues (temporal resolution, identification of biases, biases in internal versus external events) related to future use of the complication clock are interrelated. One cannot assume that the time report is a faithful representation of subjective timing and must instead ask oneself if the research question can be served by using the complication clock. Successful usage of the clock therefore involves acknowledging the limitations of the method and creating hypotheses that can accommodate the constraints inherent to the spatial nature and the potential biases of the complication clock.

A potential idea for a future study may be a metastudy similar to the one conducted by Baribault et al. (2018). In their study, Baribault et al. investigated the effect of subliminal priming on cognitive control but included many independent variables that were indiscriminately randomised. Hierarchical Bayesian modelling was used to quantify the effect size of each moderating variable used in the study. With the complication clock, such a study could be used to investigate the effect size of different biases on time reports, which could then be used to control for biases in future complication clock studies once different combinations of biases have been identified.

#### Adapting the complication clock for different theoretical orientations

The spatial form of the complication clock and the uniform motion of the pointer presents a contradiction for the study of aspects of cognition and behaviour that are increasingly considered to be dynamic in nature (Cisek & Green, 2024; Gomez-Marin & Ghazanfar, 2019). In such cases, it may be important to acknowledge that the complication clock, which by its linear form and motion of the pointer may more easily facilitate serial interpretations of cognitive phenomena, as seriality implies that different parts of a process are defined by onset/offset points, which makes it easier for time reports to investigate the temporal properties of a particular component of a serial process. This may constrain certain aspects of a theory one would like to investigate (e.g., parallel processing vs. serial processing). Furthermore, because the clock allows one to ostensibly measure *when* a mental event occurs at a single spatial location, one can be easily led to think that mental phenomena such as intention, which has also been argued to be a dynamic process (Dominik et al., 2024; Schurger & Uithol, 2015; Uithol et al., 2014), can be isolated and measured in such a discrete manner. In this way, the representation of time assumed by the clock can bias certain interpretations of cognitive phenomena (cf. the Gestalt view of mental events). A particularly illustrative example is with novel theories of action control such as Cisek’s *affordance competition hypothesis* (Cisek, 2007; Cisek & Kalaska, 2010), which we discuss here. It also highlights how one might be able to adapt the complication clock to a more dynamic theory of action control.

The classic model of action control, associated with the Libet studies, describes a serial process in which intention precedes motor planning and then execution (Dominik et al., 2024; Noel et al., 2025). This understanding of action control has mostly been based on studies where participants are only required to perform a single action in response to or alongside a single stimulus, where experimental designs conform to sequential, discrete stages (Dominik et al., 2024; Schurger & Uithol, 2015; Uithol et al., 2014). However, during natural behaviour, animals and humans perform actions in a dynamic world, wherein the structure of the environment that shapes potential actions, also known as affordances (Gibson, 1979), and their outcomes is continually evolving.

In contrast to classical serial models of action control, theories like the affordance competition hypothesis (Cisek, 2007; Cisek & Kalaska, 2010) – inspired by diverse fields such as ethology, ecological psychology, and autonomous robotics research – argue that within sensorimotor circuits, multiple potential actions compete with one another in parallel. In such a view, rather than being sequential, processes such as intention, decision, and motor planning may be dynamically intertwined and shaped by continuously evolving sensory input as well as internal goals and biases. Evidence for this theory comes from a growing body of neurophysiological data supporting the idea that the brain’s functional architecture reflects an interaction between multiple competing motor plans. For example, Cisek and Kalaska (2005) showed that when a monkey was presented with two potential reaching actions, with only one of these actions later shown to be the correct choice via a cue, neural activity in the dorsal premotor cortex specified both directions simultaneously. When the information representing the correct direction was shown, the representation of the correct direction was strengthened while the opposing direction was attenuated, reflecting resolution of multiple competing motor plans.

How might the complication clock accommodate such a theory? One issue with the complication clock is that the motion of the pointer, which serves as a spatial representation of ongoing time, moves in a linear fashion. Therefore, theories of cognition and behaviour that are serial in nature may potentially be more easily facilitated by the form of the clock. However, according to theories like the affordance competition hypothesis, multiple potential motor actions are specified and compete with one another in parallel. In such a view, rather than being sequential and linear, processes such as intention, decision, and motor planning are dynamically intertwined. This can make it difficult to reconcile the theory alongside the experimental tool. Therefore, the question becomes how one can accommodate the theory within the complication clock. One option, as suggested by Cisek and Kalaska (2010), would be to simply continue as before and to interpret data obtained from the traditional laboratory setting within a broader naturalistic context. However, this approach does not actually provide empirical evidence for the theory at hand. An alternative approach would be to incorporate aspects of the theory into complication clock studies such as the continuously evolving information environment which eventually facilitates the selection of a single motor plan. For example, Cisek et al. (2009) conducted a study in which participants observed a central circle flanked on either side with identical circles. The central circle was filled with tokens, with a token from the central circle jumping to either the right or left circle every 200 ms. Participants had to judge which circle had more tokens during the trial and move a cursor to the circle with more tokens. Importantly, the token-jumping behaviour was programmed in a manner so that the ‘evidence accumulation’ process was not perfectly reliable, with some trials intentionally programmed to be ambiguous or even tricky (i.e., the jumping behaviour of the first six tokens was meant to mislead the participant). Such an information environment could presumably be adapted for the complication clock in which participants may be asked to perform different actions (such as different keypresses) depending on the anticipated outcome. Participants can then be asked to pinpoint the time *when* they committed to their decision, similar to the study conducted by Kang et al. (2017). Such an experimental design could perhaps be used to investigate *when* competition between multiple potential actions is resolved (note, however, that this does not resolve biases of time reports in general). Although other methods may be more suitable for investigating other aspects of the affordance competition hypothesis, the example here shows that it is not impossible for the clock to investigate certain aspects of theories that are inherently dynamic. In principle, it is feasible to investigate parallel processes because the temporal properties of each individual process may be different, thus allowing one to use the clock to investigate the temporal relations between different components of a parallel process. However, to do so would require more sophisticated experimental designs and theorising into the temporal properties of different components whilst taking into account the limitations of the complication clock. Lastly, studies that have simultaneously used multiple clock stimuli (Carlson et al., 2006; Hogendoorn et al., 2007; Keetels & Vroomen, 2011) have shown that it is possible to use the complication clock in novel arrangements. For example, it may be possible to use multiple clocks in a way in that each clock could represent a different process if one were to study multiple processes simultaneously.

Addressing this issue requires more careful theorising into the nature of the specific cognitive phenomena in question as well as the nature of time perception itself. It is important to consider when the fundamental presumptions of the complication clock (seriality, discrete nature of time perception, etc.) may not hold for the theory to be tested (Cisek & Green, 2024). This approach to using the complication clock may in fact close off certain avenues of research directions if one had wanted to use the complication clock. However, it may also help researchers use the complication clock in a way that is more conducive to advancing theory development (Oberauer & Lewandowsky, 2019; Szollosi & Donkin, 2021). It is also important to consider how studies using the complication clock fit into their respective lines of research. For example, the results of the study by Kang et al.’s (2017) investigation into the temporal dimension of perceptual decision making seem to accord with the rest of the literature investigation perceptual decision making. This also seems to be the case for studies using the complication clock where there is already a large body of research behind them (such as for the attention studies – Carlson et al., 2006; Hogendoorn et al., 2007; Keetels & Vroomen, 2011; or the study by Franikowski et al., 2021). On the other hand, there are several alternative methodologies that also investigate temporal binding such as the event-anticipation paradigm (Buehner, 2012; Roth et al., 2023), which may provide a more direct readout of time perception within action-effects without the potential confound of spatial attention.

## Summary and concluding remarks

In this article, we reviewed the history of the various research programs that used the complication clock, first developed by Wilhelm Wundt to study elementary sensory processing. We showed that though the complication clock was first used to probe subjective timing into external events, the clock has undergone a shift to studying complex internal phenomena ever since Benjamin Libet introduced his landmark studies into volition and intentional action. However, underlying the history of the complication clock was an assumption that the clock was a passive measurement device of subjective time perception. It is more parsimonious to assume that the complication clock is a measure using spatial localisation, with time inferred thereafter. One can imagine that a measure of subjective timing can also be attained if an event is presented while a straight line is moving across the screen at a known speed and the position of the line during event presentation is then localised. In both cases, whatever measure of subjective time the researcher yields can only be extracted from the localised position.

We then reviewed the numerous mechanisms and biases of the complication clock such as those involving eye movements and visuospatial attention, highlighting the fact that they are inherent to the spatial form of the clock measure. The spatial form of the clock carries a further consequence for theoretical interpretation. Because the pointer traverses the clock face in a continuous, unidirectional arc, the method represents events as temporally occupying discrete, linear positions. This format is compatible with theories that describe cognitive processes as sequential stages and less suited for theories in which processes unfold concurrently or dynamically (Cisek & Green, 2024). The discrete localisation of an event on the clock face can therefore bias interpretation of cognitive phenomena even when the underlying process may not operate in such a manner (Gomez-Marin & Ghazanfar, 2019). This concern is amplified when the clock is used to time internal events such as intentions or decisions, where temporal properties of such events are not necessarily known. For external events (e.g., a tone), the physical onset is known to the experimenter and the time report can therefore be validated. However, this is not the case for internal events. The epistemic status of the time report is therefore different in the two cases, and conclusions drawn from time reports of internal events should take this asymmetry into account.

That the form of the clock can have an impact on the interpretation of experimental data and bias theory is perhaps another example of the theory-ladenness of scientific experimentation (Brewer & Lambert, 2001). Given these constraints, one way of assessing the evidential strength of clock-based findings may depend on convergence with results obtained through independent methods. Where such convergence exists (as in perceptual decision making – Kang et al., 2017; or in affect timing – Franikowski et al., 2021, corroborated by Reisenzein & Franikowski, 2022), the spatial biases of the clock can be set aside as an unlikely sole explanation. Where convergence is absent, one needs to consider the possibility that timing differences may reflect differences in the pointer localisation. The complication clock is therefore not a one-size-fits-all tool for studying cognitive phenomena which may be inherently dynamic in nature.

To conclude, we have reviewed the history and mechanisms of the complication clock and discussed avenues in which its spatial representation of time can be more precisely used in future research to investigate the temporal aspect of cognition.

## Data Availability

Not applicable.

## Footnotes

1 This reflects an error of equivocation. The authors of the early complication studies treated the relation between objective event time and subjective experience as an epistemic question. In much of the contemporary literature, by contrast, ‘objective’ is used in a methodological sense and refers to properties of measurement procedures. In that sense, time reports can be measured objectively; this is a different claim from the epistemic question regarding whether subjective report time can be identical to objective event time.

2 Further discussion of the notion of backwards referral can be found in the later discussion under “The latency of conscious awareness”.

3 Note, however, that we are not claiming that space is an unsuitable metaphor for time in general. The analogue clock, for example, has proven to be an extremely useful way of measuring time and has allowed for the social coordination of human activity even when spanning across different continents. The question is whether such a link between space and time corresponds to our psychological experience of time.
